# Comparison of the revised 4th (2016) and 5th (2022) editions of the World Health Organization classification of myelodysplastic neoplasms

**DOI:** 10.1038/s41375-022-01718-7

**Published:** 2022-10-12

**Authors:** Yudi Zhang, Junying Wu, Tiejun Qin, Zefeng Xu, Shiqiang Qu, Lijuan Pan, Bing Li, Huijun Wang, Peihong Zhang, Xin Yan, Jingye Gong, Qingyan Gao, Robert Peter Gale, Zhijian Xiao

**Affiliations:** 1 State Key Laboratory of Experimental Hematology, National Clinical Research Center for Blood Diseases, Haihe Laboratory of Cell Ecosystem, Institute of Hematology and Blood Diseases Hospital, Chinese Academy of Medical Sciences & Peking Union Medical College, Tianjin, China; 2 MDS and MPN Centre, Institute of Hematology and Blood Diseases Hospital, Chinese Academy of Medical Sciences & Peking Union Medical College, Tianjin, China; 3 Hematologic Pathology Center, Institute of Hematology and Blood Diseases Hospital, Chinese Academy of Medical Sciences & Peking Union Medical College, Tianjin, China; 4 Haematology Research Centre, Department of Immunology and Inflammation, Imperial College London, London, UK

**Keywords:** Myelodysplastic syndrome, Myelodysplastic syndrome

## Abstract

We used data from 852 consecutive subjects with myelodysplastic neoplasms (MDS) diagnosed according to the 2016 (revised 4th) World Health Organization (WHO) criteria to evaluate the 2022 (5th) edition WHO classification of MDS. 30 subjects previously classified as MDS with an *NPM1* mutation were re-classified as acute myeloid leukaemia (AML). 9 subjects previously classified as MDS-U were re-classified to clonal cytopenia of undetermined significance (CCUS). The remaining 813 subjects were diagnosed as: MDS-5q (*N* = 11 [1%]), MDS-SF3B1 (*N* = 70 [9%]), MDS-biTP53 (*N* = 53 [7%]), MDS-LB (*N* = 293 [36%]), MDS-h (*N* = 80 [10%]), MDS-IB1 (*N* = 161 [20%]), MDS-IB2 (*N* = 103 [13%]) and MDS-f (*N* = 42 [5%]) and MDS-biTP53 (*N* = 53 [7%]). 34 of these subjects came from the 53 (64%) MDS-biTP53 previously diagnosed as MDS-EB. Median survival of subjects classified as MDS using the WHO 2022 criteria was 45 months (95% Confidence Interval [CI], 34, 56 months). Subjects re-classified as MDS-biTP53 and MDS-f had significantly briefer median survivals compared with other MDS sub-types (10 months, [8, 12 months] and 15 months [8, 23 months]). In conclusion, our analyses support the refinements made in the WHO 2022 proposal.

## Introduction

A summary of the World Health Organization (WHO) 5th edition (2022) classification of myelodysplastic neoplasms (MDS) was recently published in *LEUKEMIA* [1]. The WHO 2022 classification reorganize MDS categories by emphasizing histological and genetic co-variates. Diagnostic criteria for MDS with low blasts and isolated del(5q) (MDS-5q) was unchanged. MDS with biallelic *TP53* inactivation (MDS-biTP53) is introduced as a new sub-type defined by the presence of multi-hit *TP53* mutations and supersedes other MDS sub-types. Presence of a *SF3B1* mutation and low blasts is considered consistent with a MDS diagnosis (MDS-SF3B1) and supersedes the prior entity of MDS with ring sideroblasts (MDS-RS). In the sub-types defined by histology the WHO 2022 classification retains cutoffs between MDS with low blasts (MDS-LB) and MDS with increased blasts (MDS-IB). Persons without increased blasts are divided into hypoplastic MDS (MDS-h) and MDS-LB. Persons with increase blasts are divided into MDS-IB1, MDS-IB2 and MDS with fibrosis (MDS-f). MDS-h and MDS-f are considered distinct subtypes underscoring the importance of a trephine bone marrow biopsy. We used a dataset of 852 consecutive subjects with MDS initially diagnosed using the WHO 2016 criteria to compare how these subjects would be classified using the WHO 2022. Our analyses support the refinements made in the WHO 2022 proposal.

## Subjects and methods

### Subjects

852 consecutive subjects ≥18 years with newly-diagnosed MDS according to the 2016 (revised 4th) WHO criteria in our centre from August 30, 2016 to September 22, 2021 were enrolled [2]. Bone marrow aspirate and biopsy samples were obtained from all subjects. Diagnostic procedures were according to recent recommendation [3]. Subjects were re-classified according to the WHO 2022 classification. The prognostic impact was evaluated with the International Prognostic Scoring Systems-Revised (IPSS-R) and International Prognostic Scoring Systems-Molecular (IPSS-M) [4, 5]. Baseline co-variates at diagnosis are displayed at Table 1. Follow-up data were available for 789 of subjects (93%). The last follow-up was on June 4, 2022 with a median follow-up of survivors 2 years (Inter-Quartile Range [IQR], 8–31 months). The study was approved by the Ethics Committees of the Institute of Hematology, Chinese Academy of Medical Science (CAMS) and Peking Union Medical Collage (PUMC) according to guidelines of Declaration of Helsinki.

**Table 1:** Baseline characteristics.

Characteristics	
Number of patients	852
Male, (%)	550 (64.6)
Age, years, median (IQR)	56 (44–64)
Haemoglobin, g/l, median (IQR)	79 (66–95)
WBC×10E + 9/L, median (IQR)	2.64 (1.82–3.78)
ANC×10E + 9/L, median (IQR)	1.10 (0.65–2.03)
PLT×10E + 9/L, median (IQR)	60 (31–119)
WHO 2016 classification	
MDS-SLD	49 (5.8)
MDS-MLD	365 (42.8)
MDS-RS-SLD	22 (2.6)
MDS-RS-MLD	24 (2.8)
MDS-5q-	12 (1.4)
MDS-EB1	197 (23.1)
MDS-EB2	162 (19)
MDS-U	21 (2.5)
IPSS-R karyotype (%), *n* = 760	
Very Good	10 (1.2)
Good	427 (50.1)
Intermediate	186 (21.8)
Poor	42 (4.9)
Very poor	95 (11.2)
Missing	92
IPSS-R risk group (%), *n* = 760	
Very Low	28 (3.3)
Low	185 (21.7)
Intermediate	241 (28.3)
High	175 (20.5)
Very high	131 (15.4)
Missing	92
IPSS-M risk group (%), *n* = 760	
Very Low	21 (2.5)
Low	138 (16.2)
Moderate Low	125 (14.7)
Moderate High	113 (13.3)
High	170 (20.0)
Very High	193 (25.4)
Missing	92

*WHO* World Health Organization, *MDS* myelodysplastic syndromes(neoplasms), *MDS-U* MDS unclassifiable, *SLD* single lineage dysplasia, *MLD* multilineage dysplasia, *RS-SLD* ring sideroblasts with SLD, *RS-MLD* ring sideroblasts with MLD, *EB1/2* excess blasts type 1/2, *5q-* isolated 5q deletion, *WBC* white blood cell count, *ANC* absolute neutrophil count, *PLT* platelet count, *IPSS-R* Revised International Prognostic Scoring System, *IPSS-M* International Prognostic Scoring Systems-Molecular.

### Bone marrow evaluation

Wright-Giemsa staining was done on bone marrow and blood slides for histological assessment with  ≥ , 500 and 200 nucleated cells enumerated. Prussian blue stain was done on bone marrow slides to identify and enumerate ring sideroblasts. Erythroblasts Periodic acid-Schiff (PAS), neutrophil alkaline phosphatase (N-ALP) and CD41 immune staining were done to identify dysplastic lineages as described [6, 7]. Bone marrow biopsies were done on all subjects. Routine bone marrow biopsy section thickness was 3 µm. Hematoxylin-eosin (H&E), PAS and Gomori sliver stains were done routinely. Age-adjusted bone marrow cellularity and degree of bone marrow fibrosis were determined using European consensus guidelines [8].

### Multi-parameter flow cytometry

Multi-parameter flow cytometry (MPFC) was done within 24 h after collection of EDTA-anti-coagulated bone marrow aspirates. A combination of flow antibody panel (Table S1) was designed to assess MDS associated phenotypic abnormalities according to The International and European Leukemia Net Working Group Guidelines [9].

### Cytogenetic analyses

Chromosome analyses were done on unstimulated bone marrow cells after 24 h of culture using G- and/or R-banding techniques. Chromosome identification and cytogenetic descriptors were applied following the International System for Human Cytogenetic Nomenclature [10]. In subjects with <12 metaphases we used fluorescence in situ hybridization (FISH) analyses including probes for -5/-5q, -7/-7q, +8, -20q, 17p- and -Y.

### Targeted gene sequencing

DNA from diagnosis bone marrow mononuclear cells was used for next-generation sequencing (NGS) as described [11]. We sequenced DNA from 592 subjects using a 141-genes panel from August 2016 to March 2020 (Table S2). DNA from 260 subjects was sequenced with a 267- genes panel from April 2020 to September 2021 (Table S3). *PRPF8* and *GNB1*, defined as residual genes in IPSS-M, were not included in the 141-gene panel. *TP53* allele state was determined as described [12].

### Statistics analyses

Continuous co-variates were described by median and IQR and categorical co-variates were summarized with count and relative frequency. Continuous co-variates (non-normal distribution) were compared using the Mann–Whitney U tests. Categorical co-variates were compared using the Fisher exact test or the Pearson chi-square test. Survival was calculated as the interval from diagnosis to last follow-up or death and analyzed by the Kaplan-Meier method. The log-rank test was used for uni-variable comparisons. All *P*-values were 2-tailed. Statistical significance was set at *P*  <  0.05. Analyses were conducted using GraphPad Prism 8 software (GraphPad Software, San Diego, CA, USA) and SPSS software (IBM, Chicago, IL, USA).

## Results

### Re-stratification from the WHO 2016 to WHO 2022 classifications

Diagnostic criteria were largely unchanged in the new proposal except for these: (1) persons with *KMT2A, MECOM, NUP98* rearrangements and *NPM1* mutation are classified as acute myeloid leukaemia (AML) regardless of percentage blasts; and (2) diagnosis of MDS unclassifiable (MDS-U) was eliminated and partly replaced by clonal cytopenia of undetermined significance (CCUS). Applying the WHO 2022 classification 30 subjects with an *NPM1* mutation were re-classified as AML, previously classified as MDS with excess blast type2 (MDS-EB2; *n* = 13), MDS with multi-lineage dysplasia (MDS-MLD; *n* = 9), MDS with excess blast type1 (MDS-EB1; *n* = 6) and MDS-U (*n* = 2) according to the WHO 2016 criteria. Nine subjects previously classified as MDS-U were re-classified to CCUS.

**Fig. 1:**
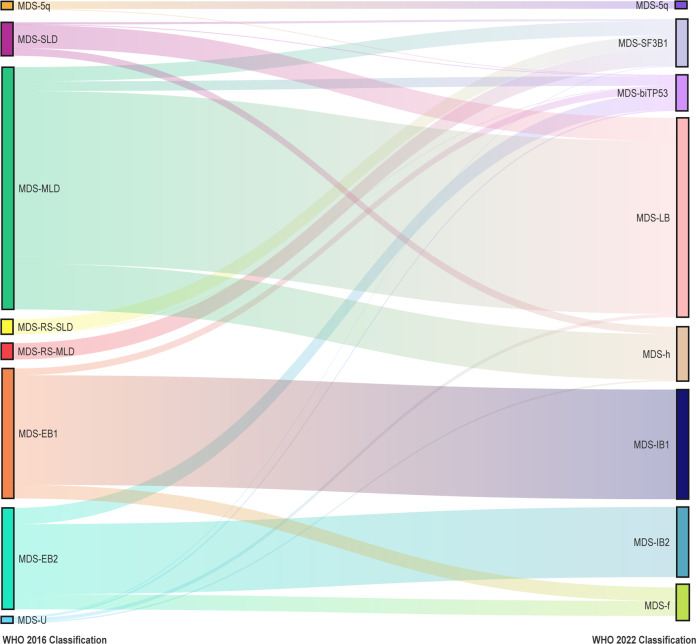
Summary of the relationship between MDS patients’ subtypes defined in the WHO 2016 and WHO 2022 classification. WHO World Health Organization, MDS myelodysplastic syndromes(neoplasms), MDS-U MDS unclassifiable, SLD single lineage dysplasia, MLD multilineage dysplasia, RS-SLD ring sideroblasts with SLD, RS-MLD ring sideroblasts with MLD, EB1/2 excess blasts type 1/2, 5q- isolated 5q deletion, biTP53 biallelic TP53 inactivation, LB low blasts, MDS-SF3B1 MDS with low blasts and SF3B1 mutation, MDS-h MDS, hypoplastic, IB1/2 increased blasts type1/2, MDS-f MDS with fibrosis.

Re-classification of remaining 813 subjects between the WHO 2016 to WHO 2022 classification are displayed in Table 2 and Fig. 1. Classification of the 11 subjects with MDS-5q remained unchanged. In addition to prior MDS-RS subjects (*N* = 45), 25 subjects without excess blasts were re-classified as MDS-SF3B1 because the new criteria have no limitation on numbers of ring sideroblasts. We re-classified 53 subjects as MDS-biTP53, most commonly those with excess blasts (34/53; 64%). Amongst subjects without the genetic abnormalities defined above, 80 previously classified as MDS-SLD/MLD or MDS-U were re-classified as MDS-h and the remaining 293 as MDS-LB. Subjects previously classified as MDS-EB1 or EB2 were re-classified as MDS IB1 (*N* = 161) or IB2 (*N* = 103) after those with MDS-f (*N* = 42) were excluded.

**Table 2:** Re-stratification matrix of the number of MDS patients classified in each of the WHO 2016 classification (column) and each of the WHO 2022 classification (row).

WHO-2016, *n*	MDS-5q	MDS-RS-SLD	MDS-RS-MLD	MDS-SLD	MDS-MLD	MDS-EB1	MDS-EB2	MDS-U	Number of patients, *n* (%)
WHO-2022, *n*									
MDS-5q	11	0	0	0	0	0	0	0	11(1.3)
MDS-SF3B1	0	21	24	3	21	0	0	1	70 (8.6)
MDS-biTP53	1	1	0	1	14	10	24	2	53 (6.5)
MDS-LB	0	0	0	34	254	0	0	5	293 (36)
MDS-h	0	0	0	11	67	0	0	2	80 (9.8)
MDS-IB1	0	0	0	0	0	161	0	0	161 (19.8)
MDS-IB2	0	0	0	0	0	0	103	0	103 (12.7)
MDS-f	0	0	0	0	0	20	22	0	42 (5.2)
Number of patients, *n* (%)	12 (1.5)	22 (2.7)	24 (3.0)	49 (6)	356 (43.8)	191 (23.5)	149 (18.3)	10 (1.2)	813

*WHO* World Health Organization, *MDS* myelodysplastic syndromes(neoplasms), *MDS-U* MDS unclassifiable, *SLD* single lineage dysplasia, *MLD* multilineage dysplasia, *RS-SLD* ring sideroblasts with SLD, *RS-MLD* ring sideroblasts with MLD, *EB1/2* excess blasts type 1/2, *5q-* isolated 5q deletion, *biTP53* biallelic TP53 inactivation, *LB* low blasts, *MDS-SF3B1* MDS with low blasts and SF3B1 mutation, *MDS-h* MDS hypoplastic, *IB1/2*: increased blasts type1/2, *MDS-f* MDS with fibrosis.

### Gene profile and risk categories according to IPSS-R and IPSS-M

**Fig. 2:**
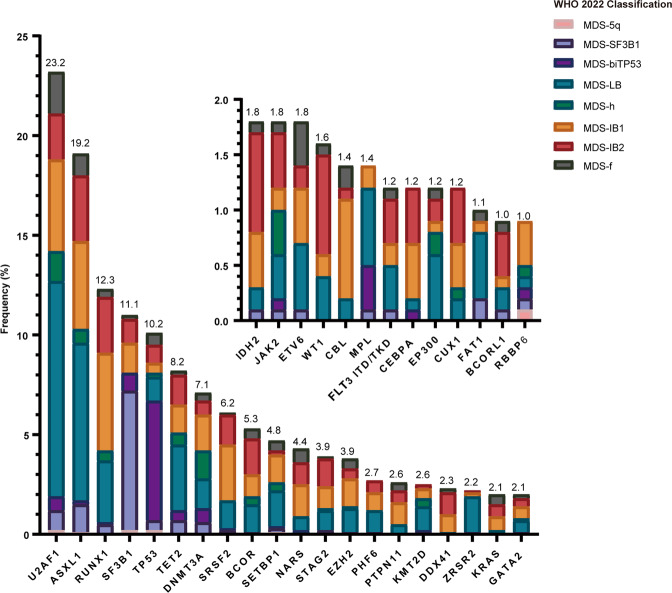
The frequency of 33 significant mutated genes (>1%) in MDS patients according to the WHO 2022 classification. WHO World Health Organization, MDS myelodysplastic syndromes(neoplasms), 5q- isolated 5q deletion, biTP53 biallelic TP53 inactivation, LB low blasts, MDS-SF3B1 MDS with low blasts and SF3B1 mutation, MDS-h MDS hypoplastic, IB1/2 increased blasts type1/2, MDS-f MDS with fibrosis.

Among the 813 subjects diagnosed as MDS using the WHO 2022 criteria, 617 subjects (76%) had ≥1 mutation including 241 subjects (30%) with 1 mutation, 167 subjects (20%) with 2 mutations and 209 subjects (26%) with ≥3 mutations. Nine genes were mutated in >5% of subjects including *U2AF1* (23%), *ASXL1* (19%), *RUNX1* (12%), *SF3B1* (11%), *TP53* (10%), *TET2* (8%), *DMNT3A* (7%), *SRSF2* (6%) and *BCOR* (5%). The distribution of mutations >1% is shown in Fig. 2.

**Fig. 3:**
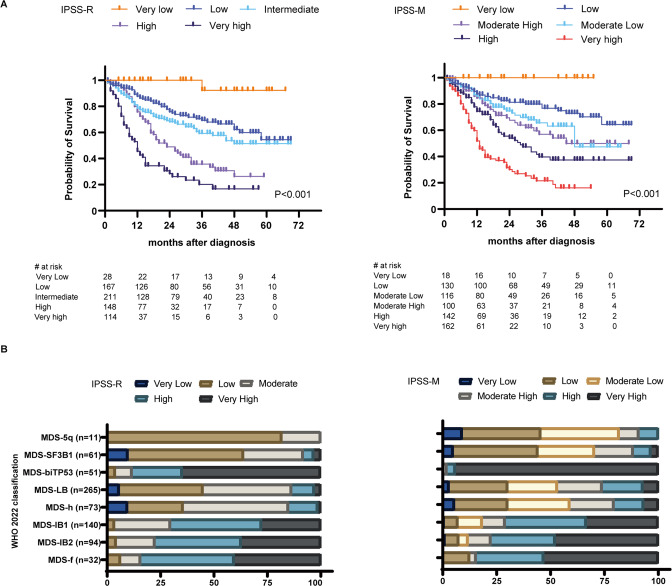
Classification and prognostication of MDS patients. Overall survival of MDS patients stratified according to the IPSS-R and IPSS-M (**A**) Repartition of IPSS-R/IPSS-M risk categories and WHO 2022 sub-types **(B)**. WHO, World Health Organization, MDS myelodysplastic syndromes(neoplasms), 5q- isolated 5q deletion, biTP53 biallelic TP53 inactivation, LB low blasts, MDS-SF3B1 MDS with low blasts and SF3B1 mutation, MDS-h MDS hypoplastic, IB1/2 increased blasts type1/2, MDS-f MDS with fibrosis, IPSS-R Revised International Prognostic Scoring System, IPSS-M International Prognostic Scoring Systems-Molecular.

The IPSS-R and IPSS-M were applied to 727 subjects (89%) with evaluable cytogenetics. Both the IPSS-R and IPSS-M were prognostically accurate in our dataset (Fig. 3A). The distribution of IPSS-R and IPSS-M risk groups differed between WHO subtypes, with the most patients allocated to high and very-high risk groups within MDS-biTP53, MDS-f and MDS-IB subsets (Fig. 3B).

### Clinical features and survival analyses of WHO 2022 MDS types

**Fig. 4:**
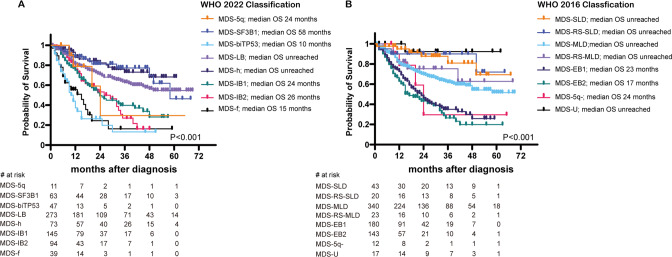
Kaplan–Meier survival curves for overall survival in MDS patients. Survival according to the WHO 2022 classification for MDS **(A)**. Survival according to the WHO 2016 classification for MDS **(B)**. WHO, World Health Organization, MDS myelodysplastic syndromes(neoplasms), MDS-U MDS unclassifiable, SLD single lineage dysplasia, MLD multilineage dysplasia, RS-SLD ring sideroblasts with SLD, RS-MLD ring sideroblasts with MLD, EB1/2 excess blasts type 1/2, 5q- isolated 5q deletion, biTP53 biallelic TP53 inactivation, LB low blasts, MDS-h MDS hypoplastic, IB1/2 increased blasts type1/2, MDS-f MDS with fibrosis, OS overall survival.

Median survival of subjects classified as MDS using the WHO 2016 criteria was 4 years (95% Confidence Interval [CI], 36, 60 months) decreasing to 45 months (34, 56 months) using the WHO 2022 criteria. Subjects with MDS-biTP53 and MDS-f in the WHO 2022 classification had significantly briefer survivals compared with other sub-types (10 months [8, 12 months] and (15 months [8, 23 months]; Fig. 4A, Table S4).

The WHO 2022 classification removes subjects with MDS-f and some with MDS-biTP53 from the WHO 2016 MDS-EB sub-type. Median survivals of MDS-IB1 (24 months [18, 30 months]) and MDS-IB2 (26 months [17, 35 months]) were significantly longer than MDS-EB1 (23 months [18, 28 months]) and MDS-EB2 (17 months [11, 23 months]; Fig. 4A, B). Subjects classified as MDS-IB1 and MDS-IB2 had similar clinical and hematological co-variates and survivals (Table S5**;** Fig. S1). Subjects with MDS-f had lower haemoglobin concentrations (75 *versus* 80 g/L; *P* = 0.02) and lower platelet concentrations (41 *versus* 61 × 10E + 9/L; *P* < 0.001). Subjects with MDS-biTP53 had lower haemoglobin concentrations (72 *versus* 80 g/L; *P* < 0.001), more complex cytogenetics (82% *versus* 14%; *P* < 0.001) and were more likely to be classified as very-high-risk in IPSS-R and IPSS-M (*P* < 0.001; Table S6) compared with subjects with MDS-IB.

Subjects with MDS-IB2 had a higher prevalence of mutations in *BCOR* (15% *versus* 6%; *P* = 0.02) and *WT1* (7% *versus* 1%; *P* = 0.03) compared with subjects with MDS-IB1. Subjects with MDS-f had a higher prevalence of *U2AF1* mutations (40% *versus* 21%; *P* = 0.01) and lower prevalence of *RUNX1* mutations (7% *versus* 24%; *P* = 0.01) compared with subjects with MDS-IB. The prevalence of mutations in *ASXL1* (4% *versus* 24%; *P* < 0.001), *RUNX1* (2% *versus* 24%; *P* < 0.001), *SRSF2* (2% *versus* 13%, *P* = 0.02), *BCOR* (0 *versus* 9%; *P* = 0.02) and *STAG2* (0 *versus* 8%; *P* = 0.03) were significantly less in subjects with MDS-biTP53 compared with those with MDS-IB.

Our subjects with MDS-h had lower concentrations of WBCs (2.40 *versus* 2.68 × 10E + 9/L; *P* = 0.004), neutrophils (1.04 *versus* 1.26×10E + 9/L; *P* = 0.004) and platelets (40 *versus* 62 × 10E + 9/L; *P* = 0.03) compared with those with MDS-LB (Table S7). Subjects with MDS-h had a significantly lower frequency of MDS-related mutations including *ASXL1* (8% *versus* 22%; *P* = 0.003) and *U2AF1* (15% *versus* 30%, *P* = 0.007) [13]. Median survivals of both cohorts were unreached but those with MDS-h had longer survival. (*P* = 0.09; Fig. 4A, and S2).

In contrast with the WHO 2016 classification the WHO 2022 classification does not distinguish numbers of dysplastic lineages. Subjects with single lineage dysplasia are at lower risk compared with those with multi-lineage dysplasia in some studies [14, 15]. We tested the prognostic value of numbers of dysplastic lineages in subjects with MDS-LB. Based on histological analyses of bone marrow slides 34 subjects were considered MDS-LB with single lineage dysplasia (MDS-LB-SLD) and 259, MDS-LB with multiple lineage dysplasia (MDS-LB-MLD). Subjects with MDS-LB-SLD had higher concentrations of haemoglobin (93 *versus* 81 g/L; *P* = 0.001), WBCs (3.80 *versus* 2.57 × 10 E + 9/L; *P* = 0.002) and neutrophils (2.14 *versus* 1.21 × 10E + 9/L; *P* = 0.001) and were more often classified as low-risk in IPSS-R (*P* = 0.003) and IPSS-M (*P* = 0.004; Table S8). Mutation patterns were similar between subjects with MDS-LB-SLD and MDS-LB-MLD. Median survivals were unreached but MDS-LB-SLD had longer survival (*P* = 0.02; Fig. S3).

## Discussion

We used data from 852 consecutive subjects with myelodysplastic neoplasms (MDS) diagnosed according to the 2016 (revised 4th) World Health Organization (WHO) criteria to evaluate the 2022 (5th) edition WHO classification of MDS. Overall, our analyses support the refinements made in the WHO 2022 proposal. Below we discuss some differences between the classifications.

*NPM1* mutations, common in AML, also occur in persons with MDS, are associated with Auer rods and can rapidly progress to AML [13, 16]. Those people were biologically more akin to AML regardless of blast percentage and may benefit from AML therapies [17, 18]. Consistent with these findings the WHO 2022 re-classifies these subjects to AML.

The category of MDS-U involves a small subset of subjects who cannot be accurately into any other MDS sub-type. Three categories in the WHO 2016 classification of this sub-type include: (1) 1% blood blasts; (2) pancytopenia and single lineage dysplasia; and (3) absence of significant dysplasia but with MDS-defining cytogenetic abnormalities [2]. People with the 1st 2 categories now re-identified as MDS-LB in the WHO 2022 classification. However, those with 1% blood blasts have a poor prognosis resembling to MDS-EB and should be closely followed [19, 20]. People in the 3^rd^ category do not fulfill current diagnostic criteria for MDS and are considered CCUS in the WHO 2022 classification. Consequently, MDS-U is removed from the WHO 2022 classification.

*SF3B1* mutations are common in people with MDS-RS [21]. The ring sideroblasts thought to be caused by impaired iron homeostasis [22, 23]. In the WHO 2016 classification persons with *SF3B1* mutation and as few as 5% ring sideroblasts without excess blasts are identified as MDS-RS [2]. Recent studies report *SF3B1* mutation identifies a homogeneous subgroup regardless of bone marrow sideroblasts or dysplasia lineages [24, 25]. For this reason the WHO 2022 classification substitutes MDS-SF3B1 for MDS-RS and incorporates single and multi-lineage dysplasia. This change may qualify more people to receive luspatercept [26].

Subjects with MDS-biTP53 and MDS-f had the briefest survivals in our dataset consistent to prior studies [12, 27–29]. Multi-hit *TP53* mutations in MDS identify a very-high-risk sub-type independent of IPSS-R and co-mutation patterns. Those persons typically have complex cytogenetics, fewer co-mutations, rapid disease progression and therapy resistance [12]. Moderate to severe bone marrow fibrosis in MDS is an independent adverse risk co-variate for more severe thrombopenia, faster progression to AML and bone marrow failure and is associated with poor survival [27–29]. We found a high frequency of *U2AF1* mutations in subjects with MDS-f. We previously reported U2AF1 mutations were associated with grades-2/-3 bone marrow fibrosis [11]. Our data suggest MDS-biTP53 and MDS-f should be recognized as distinct sub-types as in the WHO 2022 classification.

Persons with MDS-h have more severe cytopenias but a better prognosis compared with those with normal/hyper-cellular MDS [30]. These findings were reproduced in our dataset. Persons with hypoplastic MDS were reported to have specific immunological and genomic features, suggesting a unique pathogenesis of this subset [31–33]. In accordance with these findings, the WHO 2022 classification recognize MDS-h as a distinct MDS sub-type. The distinction of MDS-h and other hypoplastic bone marrow failure disorders can be difficult. Careful morphological evaluation is critical [30, 34].

Subjects with single lineage dysplasia are a small but heterogeneous cohort with a high prevalence of bi-cytopenias or pancytopenia. Isolated cytopenia is not associated with the same dysplastic lineage [35, 36]. Although persons with single lineage dysplasia were reported to have a better prognosis, replicability in identifying single *versus* multi-lineages is low [14, 15, 35, 36]. The threshold of 10% may explain these discordances and a threshold of 40% dysplastic megakaryocyte has been proposed [37]. The WHO 2022 classification integrates MDS-SLD and MDS-MLD in the WHO 2016 classification into MDS-LB in the WHO 2022 classification. The new sub-type emphasizes low blasts and provides a better description of those persons. Regardless, we suggest persons with MDS-LB-MLD may have a worse prognosis compared with people with MDS-LB-SLD.

Our study has important limitation, such as our data should be confirmed by an independent cohort.

In conclusion, our evaluation supports the refinements made in the WHO 2022 classification of MDS.

## Supplementary information

Supplemental material

## Data Availability

The datasets generated during and/or analyzed during the current study are available from the corresponding author on reasonable request.
