# Supplementary material for: Comparison of the revised 4th (2016) and 5th (2022) editions of the World Health Organization classification of myelodysplastic neoplasms
Source: Leukemia. 2022 Oct 12;36(12):2875–82. doi: 10.1038/s41375-022-01718-7 (PMC9712101; doi:10.1038/s41375-022-01718-7)
Supplement: Supplementary file 1 — Supplemental material [file 41375_2022_1718_MOESM1_ESM.pdf]

Supplementary

Supplement Table 1. The combination of flow cytometry antibody panel.

Tube1	CD16-FITC	CD117-PE	CD34-PerCPCy5.5	CD38-PECy7	CD13-APC	HLA-DR-APCCy7	CD11b-V450	CD45-V500
Tube2	CD15-FITC	CD5-PE	CD34-PerCPCy5.5	CD2-PECy7	CD7-APC	CD3-V450	CD45-V500	-
Tube3	CD35-FITC	CD16-PE	CD4-PerCPCy5.5	CD14-PECy7	CD33+CD8-APC	HLA-DR-APCCy7	CD64-V450	CD45-V500
Tube4	CD36-FITC	CD105-PE	CD56-PerCPCy5.5	CD117-PECy7	CD71-APC	CD10-APCCy7	CD19-V450	CD45-V500

Supplement Table 2. List of 141 genes included in the targeted sequencing panel.

ABL1	ANKRD26	ARID1A	ASXL1	ASXL2	ATG2B	ATM	B2M	BCL2	BCL6
BCOR	BCORL1	BIRC3	BRAF	BRINP3	BTK	CALR	CARD11	CASP8	CBL
CCND1	CCND2	CCND3	CCR4	CD28	CD58	CD798	CDC25C	CDKN1B	CDKN2A
CEBPA	CNOT3	CREBBP	CRLF2	CSF3R	CSNK1A1	CUX1	CXCR4	DDX3X	DDX41
DIS3	DNM2	DNMT3A	DNMT3B	EED	EGR1	EP300	ETNK1	ETV6	EZH2
FAM46C	FAT1	FBXW7	FGFR3	FLT3	GATA1	GATA2	GATA3	GNA13	ID3
IDH1	IDH2	IKZF1	IL7R	IRF4	JAK1	JAK2	JAK3	KDM6A	KIT
KLF2	KMT2A	KMT2D	KRAS	MAP2K1	MAPK1	MAX	MED12	MEF2B	MPL
MYC	MYD88	NF1	NOTCH1	NOTCH2	NPM1	NRAS	NT5C2	PAX5	PDGFRB
PHF6	PIGA	PLCG1	PLCG2	PPM1D	PRDM1	PRKCB	PRPS1	PTEN	PTPN11
RAD21	RBBP6	RELN	RHOA	RPL10	RUNX1	SETBP1	SETD2	SF1	SF3B1
SH2B3	SMC1A	SMC3	SPEN	SRP72	SRSF2	STAG2	STAT3	STAT5B	SUZ12
TAL1	TCF3	TERT	TET2	TNFAIP3	TNFRSF14	TP53	TPMT	TRAF3	U2AF1
USP7	WHSC1	WT1	XPO1	ZBTB7A	ZMYM3	ZRSR2	NOTCH3	NOTCH4	PRPF8
ZNF384									

Supplement Table 3. List of 267 genes included in the targeted sequencing panel.

ABCB1	ABL1	ANKRD26	APC	ARID1A	ARID1B	ARID2	ARID5B	ASXL1	ASXL2
ATG2B	ATM	ATRX	B2M	BACH2	BCL10	BCL2	BCL6	BCL7A	BCOR
BCORL1	BIRC3	BLM	BPGM	BRAF	BRCA1	BRCA2	BRIP1	BTG1	BTG2
BTK	CALR	CARD11	CBL	CBLB	BCLC	CCND1	CCND3	CCR4	CD28
CD58	CD79A	CD79B	CDC25C	CDKN1A	CDKN1B	CDKN2A	CDKN2B	CDKN2C	CEBPA
CHD2	CHD8	CIITA	CNOT3	CREBBP	CRLF2	CSF1R	CSF3R	CSMD1	CSNK1A1
CTCF	CUX1	CXCR4	CYLD	DDX3X	DDX41	DIS3	DKC1	DNM2	DNMT3A
DNMT3B	DTX1	DUSP2	EBF1	EED	EGFR	EGLN1	EGR1	ELANE	EP300
EPHA7	EPOR	ETNK1	ETV6	EZH2	FAM46C	FAS	FAT1	FAT4	FBXO11
FBXW7	FGFR3	FLT3	FOXO1	FYN	GAB2	GATA1	GATA2	GATA3	GFI1
GNA13	GNAI2	GNAS	GNB1	GSKIP	H1-2	H1-3	H1-4	H1-5	HAX1
HLA-A	HLA-C	HLA-DMB	HNRNPK	HRAS	HUWEI	HVCN1	ID3	IDH1	IDH2
IGLL5	IKZF1	IKZF2	IKZF3	IL7R	IRF2BP2	IRF4	IRF8	ITPKB	JAK1
JAK2	JAK3	JUNB	KDM6A	KIT	KLF2	KLHL6	KMT2A	KMT2B	KMT2C
KMT2D	KRAS	KRT20	LCOR	LMO2	LTB	LYN	MAP2K1	MAPK1	MAX
MCL1	MED12	MEF2B	MFHAS1	MPL	MTOR	MYC	MYCN	MYD88	MYOM2
NF1	NFE2	NFKBIA	NFKBIE	NOTCH1	NOTCH2	NOTCH3	NOTCH4	NPM1	NRAS
NT5C2	P2RY8	PALB2	PAX5	PDGFRA	PDGFRB	PDS5B	PHF6	PIGA	PIK3CA
PIK3CD	PIK3R1	PIM1	PIM2	PLCG1	PLCG2	POT1	PPM1D	PRDM1	PRF1
PRKCB	PRKD2	PRKDC	PRPF8	PRPS1	PSMB5	PTEN	PTPN1	PTPN11	PTPRD
RAD12	RASA2	RB1	RBBP6	RELN	RHOA	RPL10	RRAGC	RUNX1	SAMHD1
SBDS	SETBP1	SETD1B	SETD2	SETDB1	SF1	SF3B1	SGK1	SH2B3	SH2D1A
SMARCA4	SMC1A	SMC3	SMO	SOCS1	SP140	SPEN	SRP72	SRSF2	STAG2
STAT3	STAT5B	STAT6	SUFU	SUZ12	SYK	TAL1	TBL1XR1	TCF3	TERC
TERT	TET1	TET2	TMEM30A	TMSB4X	TNFAIP3	TNFRSF14	TOX	TP53	TPMT
TRAF3	U2AF1	UBE2A	UBR5	USP7	VAV1	VHL	WHSC1	WT1	XBP1
XPO1	ZAP70	ZBTB7A	ZFP36L1	ZMYM3	ZNF292	ZRSR2			

Supplement Table 4. Pairwise comparison of survival rates between WHO 2022 subtypes in MDS.

P value	MDS-5q	MDS-SF3B1	MDS-biTP53	MDS-LB	MDS-h	MDS-IB1	MDS-IB2
**MDS-5q**	-	-	-	-	-	-	-
**MDS-SF3B1**	0.208	-	-	-	-	-	-
**MDS-biTP53**	0.038	<0.001		-	-	-	-
**MDS-LB**	0.389	0.237	<0.001	-	-	-	-
**MDS-h**	0.060	0.754	<0.001	0.094	-	-	-
**MDS-IB1**	0.702	<0.001	<0.001	<0.001	<0.001	-	-
**MDS-IB2**	0.685	<0.001	<0.001	<0.001	<0.001	0.707	-
**MDS-f**	0.073	<0.001	0.548	<0.001	<0.001	0.002	0.004

Abbreviations: WHO, World Health Organization; MDS: myelodysplastic syndromes(neoplasms); 5q-: isolated 5q deletion; biTP53: biallelic TP53 inactivation; LB: low blasts; MDS-h: MDS, hypoplastic; IB1/2: increased blasts type1/2; MDS-f: MDS with fibrosis.

Supplement Table 5. Clinical and laboratory characteristics of MDS-IB1 and MDS-IB2 patients

**Characteristics**	**MDS-IB1 (n=161)**	**MDS-IB2 (n=103)**	**P value**
Male, (%)	117 (73)	73 (71)	0.780
Age, years, median (IQR)	59 (48-65)	59 (52-65)	0.618
Haemoglobin, g/l, median (IQR)	80 (67-98)	75 (60-87)	0.467
WBC×10E+9/L, median (IQR)	2.40 (1.63-3.28)	2.25 (1.53-3.94)	0.825
ANC×10E+9/L, median (IQR)	0.88 (0.52-1.79)	0.74 (0.37-1.88)	0.375
PLT×10E+9/L, median (IQR)	61 (37-100)	41 (18-56)	0.090
IPSS-R karyotype (%)			
Very Good	3 (2.1)	2 (2.1)	
Good	71 (50.4)	59 (62.8)	
Intermediate	42 (29.8)	22 (23.4)	0.449
Poor	8 (5.7)	3 (3.2)	
Very poor	17 (12.1)	8 (8.5)	
Normal karyotype (%)	69 (48.9)	55 (58.5)	0.182
Complex karyotype (%)	22 (15.6)	10 (10.6)	0.334
IPSS-R risk group (%)			
Very Low	0	0	
Low	5 (3.5)	4 (4.3)	0.326
Intermediate	37 (26.2)	17 (18.1)	
High	60 (42.6)	38 (40.4)	
Very High	39 (27.7)	35 (37.2)	
IPSS-M risk group (%)			
Very Low	1 (0.7)	1 (1.1)	
Low	9 (6.4)	6 (6.4)	
Moderate low	16 (11.3)	4 (4.3)	0.189
Moderate High	15 (10.6)	10 (10.6)	
High	53 (37.6)	28 (29.8)	
Very High	47 (33.3)	45 (47.9)	

Abbreviations: MDS: myelodysplastic syndromes(neoplasms); IB1/2: increased blasts type1/2; WBC, white blood cell count; ANC, absolute neutrophil count; PLT, platelet count; IPSS-R, Revised International Prognostic Scoring; IPSS-M, International Prognostic Scoring Systems-Molecular.

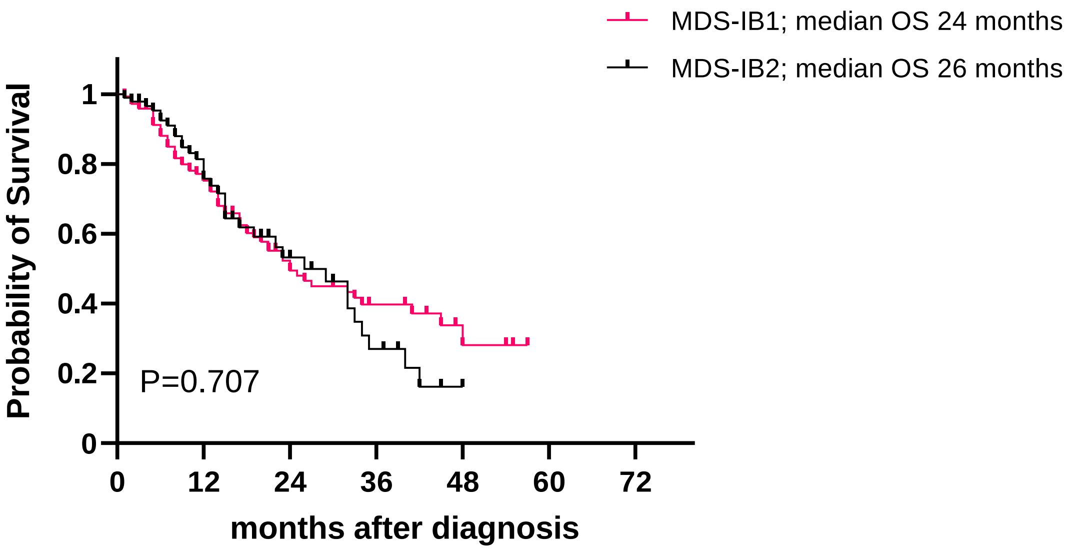

# at risk						
MDS-IB1	145	79	37	17	6	0
MDS-IB2	94	43	17	7	1	0

Supplement Figure1. Kaplan–Meier curves for Overall Survival in MDS-IB1 and MDS-IB2 patients.

Median OS for MDS-IB1 and MDS-IB patients were 24 and 26 months, respectively. Survival rate between MDS-IB1 and MDS-IB2 patients were comparable(P=0.707).

Abbreviations: MDS: myelodysplastic syndromes(neoplasms); IB1/2: increased blasts type1/2; OS: overall survival.

Supplement Table 6. Clinical and laboratory characteristics of MDS-IB, MDS-biTP53 and MDS-f patients

Characteristics	MDS-IB* (n=264)	MDS-f (n=42)	MDS-biTP53 (n=53)	P1	P2	P3
Male, (%)	190 (72)	30 (71.4)	32 (60.4)	0.720	0.064	0.286
Age, years, median (IQR)	59 (48-65)	59 (52-65)	60 (54-64)	0.867	0.516	0.618
Haemoglobin, g/l, median (IQR)	80 (67-98)	75 (60-87)	72 (59-82)	0.023	<0.001	0.467
WBC×10E+9/L, median (IQR)	2.40 (1.63-3.28)	2.25 (1.53-3.94)	2.82 (1.70-3.57)	0.772	0.323	0.825
ANC×10E+9/L, median (IQR)	0.88 (0.52-1.79)	0.74 (0.37-1.88)	1.04 (0.49-2.02)	0.333	0.236	0.375
PLT×10E+9/L, median (IQR)	61 (37-100)	41 (18-56)	45 (29-108)	<0.001	0.207	0.090
IPSS-R karyotype (%)						
Very Good	5 (2.1)	0	0			
Good	130 (55.3)	14 (43.8)	5 (9.8)			
Intermediate	64 (27.2)	10 (31.3)	2 (3.9)	0.487	<0.001	<0.001
Poor	11 (4.7)	2 (6.3)	4 (7.8)			
Very poor	25 (10.6)	6 (18.8)	40 (78.4)			
Normal karyotype (%)	124 (52.8)	11 (34.4)	3 (5.9)	0.060	<0.001	0.001
Complex karyotype (%)	32 (13.6)	6 (18.8)	42 (82.4)	0.423	<0.001	<0.001
IPSS-R risk group (%)						
Very Low	0	0	0			
Low	9 (3.8)	2 (6.3)	2 (3.9)	0.227	<0.001	0.150
Intermediate	54 (23)	3 (9.4)	4 (7.8)			
High	98 (41.7)	14 (43.8)	12 (23.5)			
Very High	74 (31.5)	13 (40.6)	33 (64.7)			
IPSS-M risk group (%)						
Very Low	2 (0.9)	0	0			
Low	15 (6.4)	4 (12.5)	0			
Moderate low	20 (8.5)	0	0	0.183	<0.001	<0.001
Moderate High	25 (10.6)	1 (3.1)	1 (2)			
High	81 (34.5)	10 (31.3)	2 (3.9)			
Very High	92 (39.1)	17 (53.1)	48 (94.1)			

* MDS-IB1 and MDS-IB2 were merged into MDS-IB

p1, p value for MDS-IB vs. MDS-f; p2, value for MDS-IB vs. MDS-biTP53; p3, p value for MDS-f vs. MDS-biTP53.

Abbreviations: MDS: myelodysplastic syndromes(neoplasms); biTP53: biallelic TP53 inactivation; IB1/2: increased blasts type1/2; MDS-f: MDS with fibrosis; WBC, white blood cell count; ANC, absolute neutrophil

count; PLT, platelet count; IPSS-R, Revised International Prognostic Scoring System; IPSS-M, International Prognostic Scoring Systems-Molecular.

Supplement Table 7. Clinical and laboratory characteristics of MDS-LB and MDS-h patients

**Characteristics**	**MDS-LB (n=293)**	**MDS-h (n=80)**	**P value**
Male, (%)	181 (61.8)	54 (67.5)	0.364
Age, years, median (IQR)	52 (40-62)	52 (38-60)	0.543
Haemoglobin, g/l, median (IQR)	82 (67-99)	78 (65-98)	0.343
WBC×10E+9/L, median (IQR)	2.68 (1.96-4.00)	2.40 (1.76-3.29)	0.004
ANC×10E+9/L, median (IQR)	1.26 (0.81-2.16)	1.04 (0.66-1.59)	0.004
PLT×10E+9/L, median (IQR)	62 (32-131)	40 (23-80)	0.027
IPSS-R karyotype (%)			
Very Good	3 (1.1)	1 (1.4)	
Good	157 (59.2)	43 (58.9)	
Intermediate	72 (27.3)	20 (27.4)	0.107
Poor	13 (4.9)	8 (11)	
Very poor	20 (7.5)	1 (4.8)	
Normal karyotype (%)	137 (51.7)	41 (56.2)	0.511
Complex karyotype (%)	30 (11.3)	8 (11)	1.000
IPSS-R risk group (%)			
Very low	15 (5.7)	7 (9.6)	
Low risk	104 (39.2)	19 (26)	0.182
Intermediate	110 (41.5)	36 (49.3)	
High	28 (10.6)	10 (13.7)	
Very high	8 (3.0)	1 (1.4)	
IPSS-M risk group (%)			
Very Low	8 (3)	4 (5.5)	
Low	72 (27.2)	18 (24.7)	
Moderate low	61 (23)	21 (28.8)	0.737
Moderate High	55 (20.8)	15 (20.5)	
High	50 (18.9)	10 (13.7)	
Very High	19 (7.2)	5 (6.8)	

Abbreviations: MDS: myelodysplastic syndromes(neoplasms); LB: low blasts; MDS-h: MDS, hypoplastic; WBC, white blood cell count; ANC, absolute neutrophil count; PLT, platelet count; IPSS-R, Revised International Prognostic Scoring System; IPSS-M, International Prognostic Scoring Systems-Molecular.

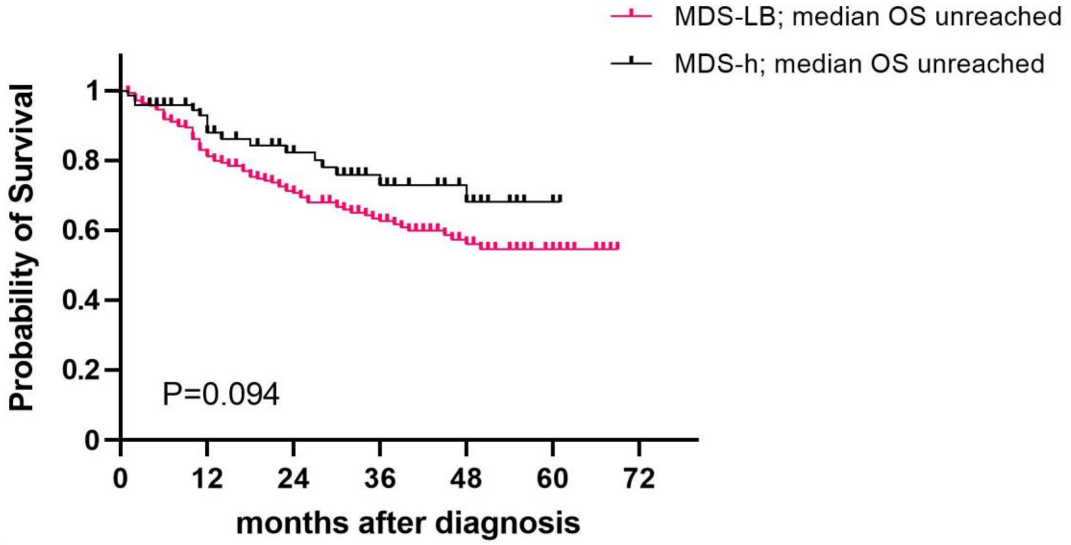

# at risk						
MDS-LB	273	181	109	71	43	14
MDS-h	73	57	40	26	15	4

Supplement Figure2. Kaplan–Meier curves for Overall Survival in MDS-LB and MDS-h patients.

Median OS for both MDS-LB and MDS-h were unreached. MDS-h patients had a trend of better prognosis compared with MDS-LB patients(P=0.094).

Abbreviations: MDS: myelodysplastic syndromes(neoplasms); LB: low blasts; MDS-h: MDS, hypoplastic; OS: overall survival.

Supplement Table 8. Clinical and laboratory characteristics of MDS-LB-SLD and MDS-LB-MLD patients.

**Characteristics**	**MDS-LB-SLD (n=34)**	**MDS-LB-MLD (n=259)**	**P value**
Male, (%)	18 (52.9)	163 (62.9)	0.266
Age, years, median (IQR)	58 (44-62)	51(40-62)	0.413
Haemoglobin, g/l, median (IQR)	93 (80-120)	81 (66-98)	0.001
WBC×10E+9/L, median (IQR)	3.80 (2.60-4.92)	2.57 (1.91-3.84)	0.002
ANC×10E+9/L, median (IQR)	2.14 (1.22-3.04)	1.21 (0.79-1.99)	0.001
PLT×10E+9/L, median (IQR)	60 (31-133)	62 (32-131)	0.950
IPSS-R karyotype (%)			
Very Good	0	3 (1.3)	
Good	20 (66.7)	137 (58.3)	
Intermediate	7 (23.3)	65 (27.7)	0.590
Poor	0	13 (5.5)	
Very poor	3 (10)	17 (7.2)	
Normal karyotype (%)	17 (56.7)	115 (51.5)	0.699
Complex karyotype (%)	3 (10)	27 (11.5)	1.000
IPSS-R risk group (%)			
Very low	6 (20)	9 (3.8)	
Low risk	14 (46.7)	90 (38.3)	0.003
Intermediate	7 (23.3)	103 (43.8)	
High	2 (6.7)	26 (11.1)	
Very high	1 (3.3)	7 (3)	
IPSS-M risk group (%)			
Very Low	3 (10)	5 (2.1)	
Low	15 (50)	57 (24.3)	
Moderate low	5 (16.7)	56 (23.8)	0.004
Moderate High	4 (13.3)	51 (21.7)	
High	3 (10)	47 (20)	
Very High	0	19 (8.1)	

Abbreviations: MDS: myelodysplastic syndromes(neoplasms); LB-SLD/MLD: low blasts with single lineage dysplasia/ multilineage dysplasia; WBC, white blood cell count; ANC, absolute neutrophil count; PLT, platelet count; IPSS-R, Revised International Prognostic Scoring System; IPSS-M, International Prognostic Scoring Systems-Molecular.

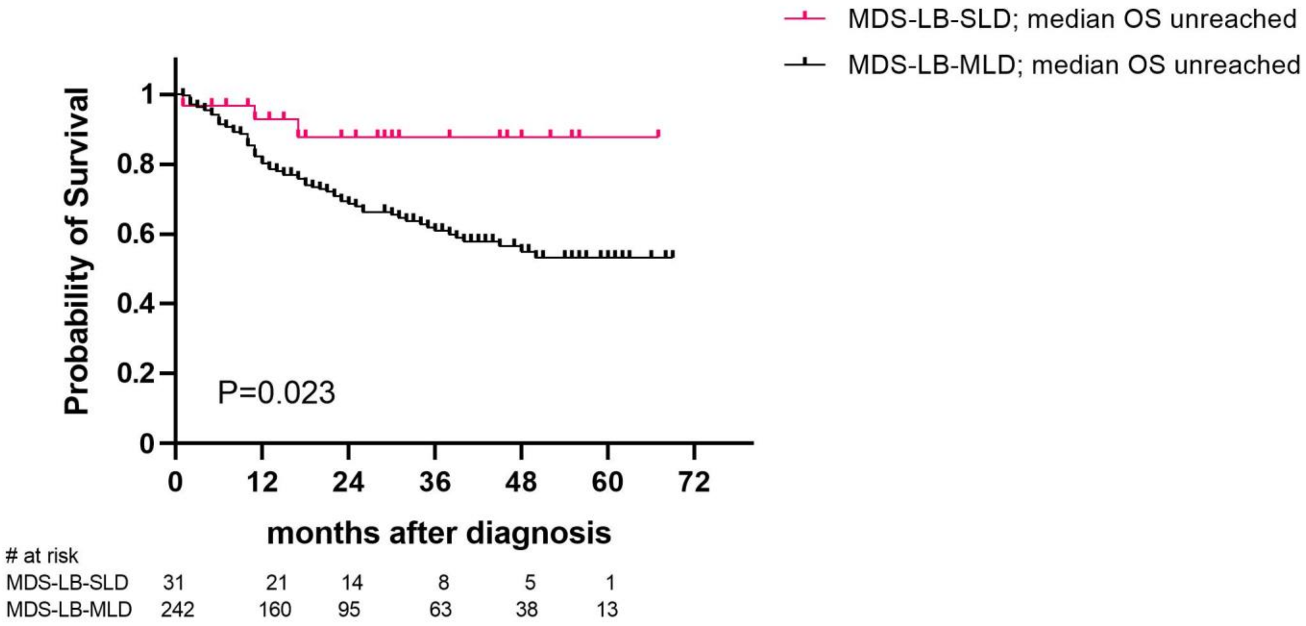

Supplement Figure3. Kaplan–Meier curves for Overall Survival in MDS-LB-SLD and MDS-LB-MLD patients.

Median OS for both MDS-LB-SLD and MDS-LB-MLD were unreached. MDS-LB-SLD patients had a better prognosis compared with MDS-LB-MLD patients(P=0.023).

Abbreviations: MDS: myelodysplastic syndromes(neoplasms); LB: low blasts; SLD: single lineage dysplasia; MLD: multilineage dysplasia; OS: overall survival.
